# Supplementary material for: Breeders that receive help age more slowly in a cooperatively breeding bird
Source: Nat Commun. 2019 Mar 21;10:1301. doi: 10.1038/s41467-019-09229-3 (PMC6428877; doi:10.1038/s41467-019-09229-3)
Supplement: Supplementary file 1 — Supplementary Information [file 41467_2019_9229_MOESM1_ESM.pdf]

Supplementary Information

Breeders that receive help age more slowly in a cooperatively breeding bird

Hammers *et al.*

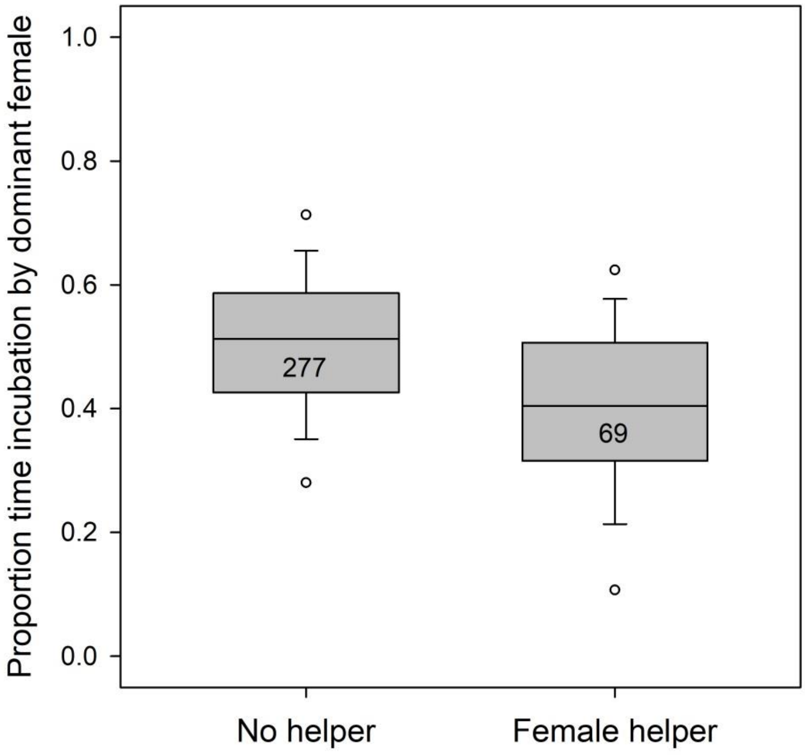

Supplementary Figure 1. Incubation attendance by dominant females in relation to helper presence. Boxplots show median and 5%, 10%, 25%, 75%, 90% and 95% quantiles. Numbers are sample sizes. Source data are provided as a Source Data file.

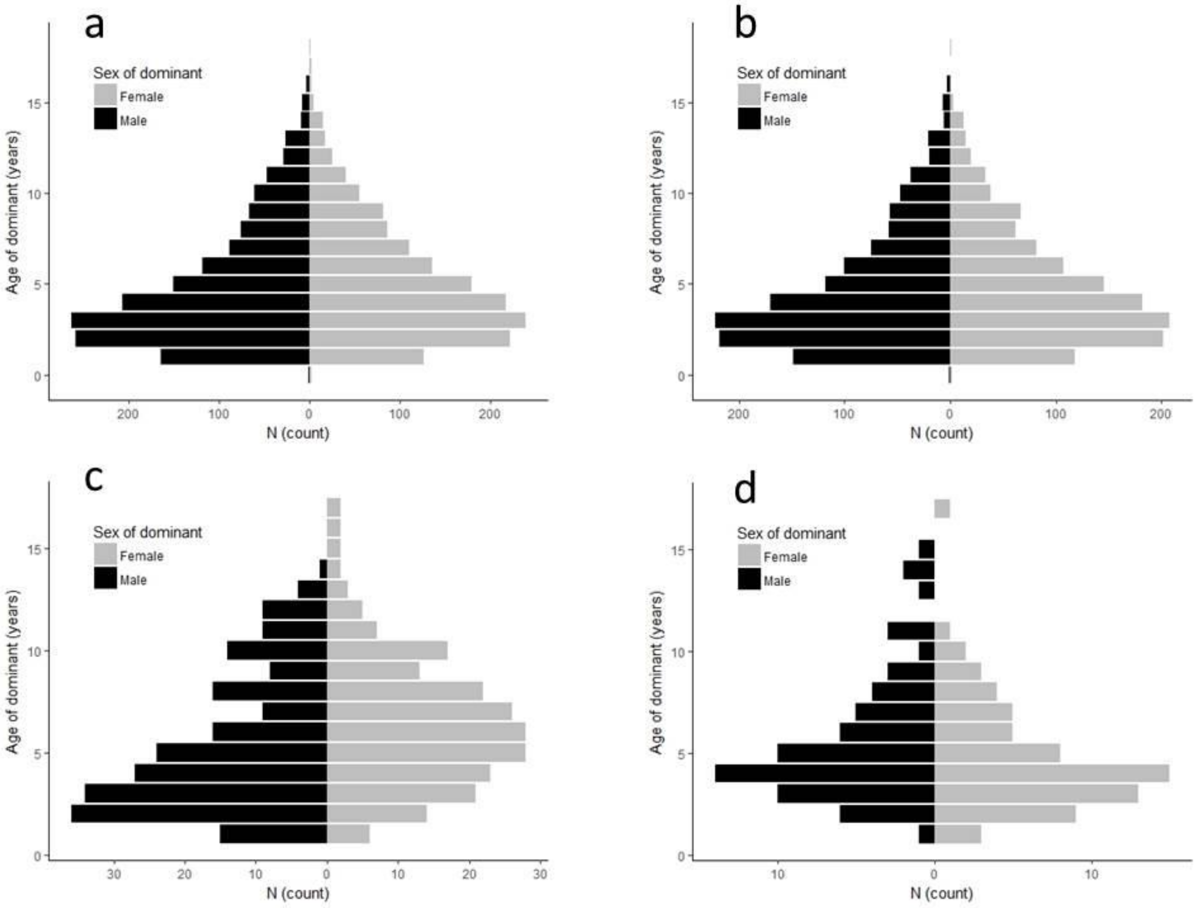

Supplementary Figure 2. Age distribution of female and male dominants. (a) irrespective of helper presence; (b) without helpers; (c) with female helpers; (d) with male helpers. Source data are provided as a Source Data file.

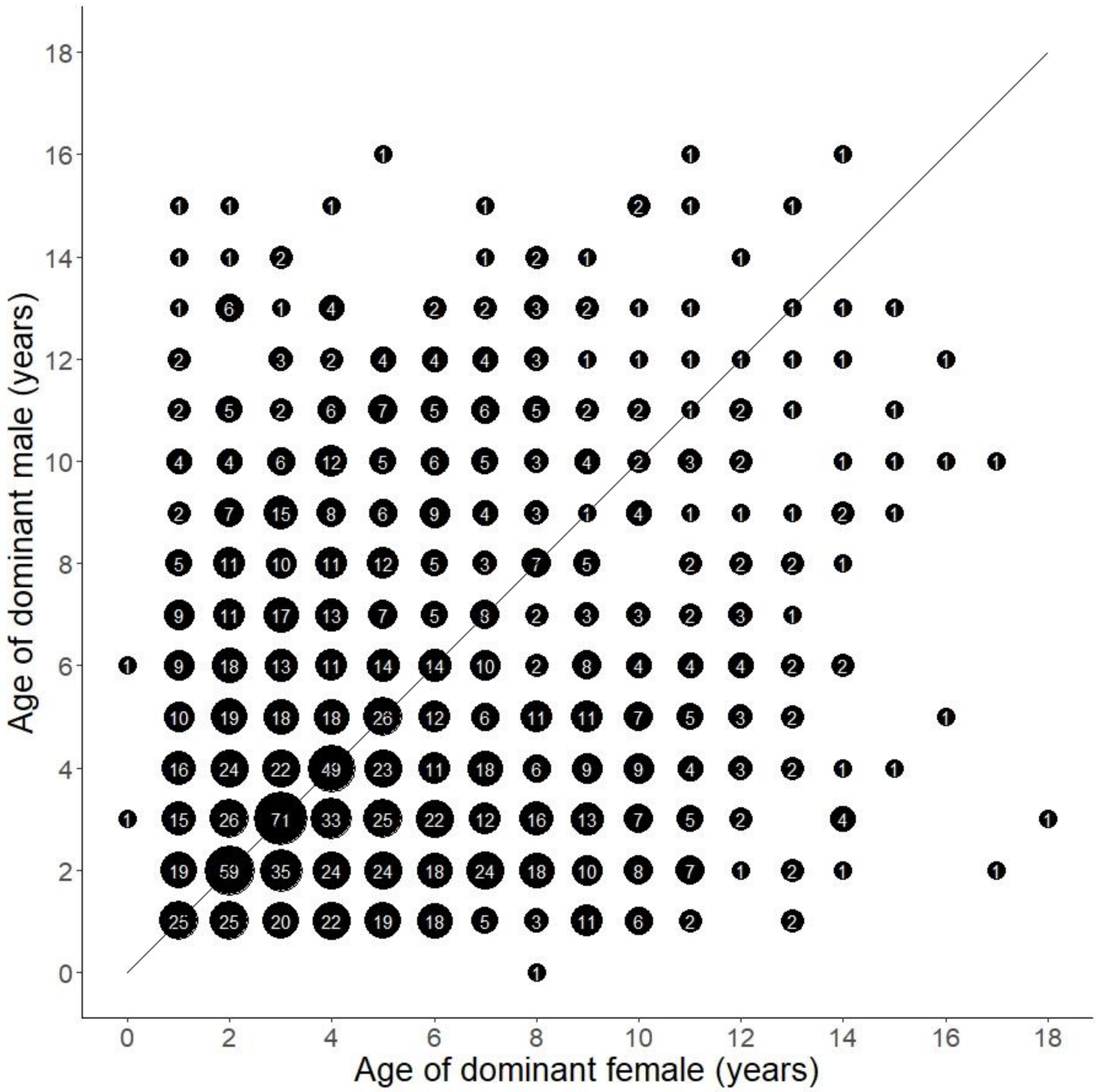

Supplementary Figure 3. The ages of the male and the female dominant within a pair. The x = y slope is given for reference. The size of the date points is proportional to the number of occurrences of each combination of male and female age. Numbers are sample sizes. Source data are provided as a Source Data file.

Supplementary Table 1. Incubation attendance in relation to helper presence and age.

(a) Incubation attendance dominant female					(b) Incubation attendance all incubating females			
**Variable**	**Estimate**	**SE**	***t***	***P***	**Estimate**	**SE**	***t***	***P***
Intercept	0.50	0.02	30.23	0.000	0.50	0.02	29.89	<0.001
Age	-0.01	0.01	-0.49	0.626	-0.01	0.02	-0.45	0.652
Age^2	0.04	0.05	0.66	0.510	0.06	0.06	1.06	0.292
Territory quality	0.00	0.02	0.09	0.932	0.00	0.02	0.01	0.992
Helper (Y/N)	-0.10	0.02	-4.97	<0.001	0.23	0.02	10.91	<0.001
Number of subordinates	0.00	0.02	0.11	0.911	-0.01	0.02	-0.35	0.726
Age x helper	0.02	0.04	0.46	0.647	0.02	0.04	0.55	0.584
Age x number of subordinates	0.00	0.02	0.26	0.797	-0.01	0.03	-0.15	0.883
Random	**Variance**				**Variance**			
Individual ID	<0.01		346 observations		<0.01		346 observations	
Year	<0.01		192 individuals		<0.01		192 individuals	
Residual	0.02		12 years		0.02		12 years	

(a) incubation attendance by dominant females, (b) incubation attendance by all incubating females (dominants and female helpers). Final models contained all main effects.

Supplementary Table 2. Age-dependent survival probability in relation to female and male helper presence.

(a) Dominant female					(b) Dominant male			
**Variable**	**Estimate**	**SE**	***z***	***P***	**Estimate**	**SE**	***z***	***P***
Intercept	1.98	0.14	14.07	<0.001	1.75	0.17	10.26	<0.001
Age	-0.51	0.25	-2.04	0.041	-0.09	0.19	-0.49	0.623
Age^2	-0.64	0.22	-2.87	0.004	-0.59	0.22	-2.71	0.007
Territory quality	0.41	0.17	2.44	0.015	0.06	0.16	0.35	0.725
Female helper (Y/N)	-0.04	0.27	-0.16	0.876	0.23	0.23	1.00	0.315
Male helper (Y/N)	-0.63	0.34	-1.84	0.066	0.30	0.36	0.83	0.407
Number of subordinates	0.26	0.19	1.34	0.179	-0.26	0.16	-1.66	0.098
Age x female helper	0.94	0.41	2.28	0.023	0.76	0.38	1.97	0.049
Age x male helper	0.63	0.74	0.85	0.398	-0.18	0.64	-0.28	0.778
Age x number of subordinates	-0.02	0.36	-0.05	0.959	-0.13	0.28	-0.46	0.649
Random	**Variance**		1571 observations		**Variance**		1581 observations	
Individual ID	0.21		463 individuals		<0.01		491 individuals	
Year	0.10		15 years		0.29		15 years	

(a) dominant females, (b) dominant males. Final models contained all main effects and significant interaction terms.

Supplementary Table 3. Annual change in relative telomere length (RTL) in relation to the presence of helpers of either sex.

(a) Dominant female					(b) Dominant male			
**Variable**	**Estimate**	**SE**	***t***	***P***	**Estimate**	**SE**	***t***	***P***
Intercept	-0.21	0.04	-4.73	<0.001	-0.07	0.07	-0.95	0.351
Initial RTL	-0.78	0.07	-11.90	<0.001	-0.56	0.08	-6.74	<0.001
Age	0.10	0.07	1.46	0.154	-0.08	0.09	-0.86	0.397
Territory quality	-0.02	0.06	-0.26	0.794	0.12	0.08	1.46	0.150
Offspring produced (Y/N)	-0.01	0.07	-0.20	0.847	0.08	0.08	0.96	0.341
Helper (Y/N)	0.50	0.12	4.27	<0.001	0.03	0.10	0.25	0.802
Number of subordinates	-0.37	0.09	-3.99	<0.001	-0.01	0.09	-0.12	0.905
Random	**Variance**		45 observations		**Variance**		74 observations	
Individual ID	<0.01		39 individuals		0.02		58 individuals	
Cohort	<0.01		18 cohorts		0.02		16 cohorts	
Year	<0.01		11 years		<0.01		9 years	
Residual	0.04				0.07			

(a) Dominant females, (b) dominant males. Final models contained all main effects.

Supplementary Table 4. The likelihood that a female subordinate reproduced (co-breeding) in relation to the age of the dominants.

(a) Dominant female					(b) Dominant male			
**Variable**	**Estimate**	**SE**	***z***	***P***	**Estimate**	**SE**	***z***	***P***
Intercept	-4.13	0.53	-7.84	<0.001	-3.87	0.45	-8.56	<0.001
Dominant age	0.01	0.05	0.24	0.808	0.00	0.04	-0.05	0.961
Territory quality	0.45	0.35	1.26	0.207	0.21	0.30	0.72	0.473
Subordinate age	3.10	0.44	7.10	<0.001	2.97	0.41	7.21	<0.001
Number of subordinates	0.40	0.30	1.36	0.174	0.22	0.27	0.80	0.422
Random	**Variance**		571 observations		**Variance**		569 observations	
Group	<0.01		465 groups		<0.01		464 groups	
Individual ID	0.3		226 individuals		<0.01		239 individuals	
Year	0.16		15 years		0.09		15 years	

(a) Dominant females, (b) dominant males. Final models contained all main effects.

Supplementary Table 5. Relative telomere length (RTL) in dominant Seychelles warblers in relation to the presence of a helper of either sex.

(a) Dominant female					(b) Dominant male			
**Variable**	**Estimate**	**SE**	***t***	***P***	**Estimate**	**SE**	***z***	***P***
Intercept	0.93	0.07	12.48	<0.001	0.84	0.05	15.48	<0.001
Age	-0.17	0.12	-1.48	0.148	0.07	0.08	0.88	0.385
Territory quality	-0.02	0.12	-0.20	0.846	-0.06	0.07	-0.84	0.403
Helper (Y/N)	-0.28	0.20	-1.37	0.178	-0.07	0.10	-0.77	0.443
Number of subordinates	0.26	0.15	1.71	0.096	0.11	0.08	1.41	0.164
Random	**Variance**		45 observations		**Variance**		74 observations	
Individual ID	<0.01		39 individuals		<0.01		58 individuals	
Cohort	<0.01		18 cohorts		0.02		16 cohorts	
Year	0.02		11 years		<0.01		9 years	
Residual	0.13				0.08			

(a) Dominant females, (b) dominant males. Final models contained all main effects.

Supplementary Table 6. Pre-breeding (July) body mass in dominants in relation to helper presence.

(a) Dominant female					(b) Dominant male			
**Variable**	**Estimate**	**SE**	***t***	***P***	**Estimate**	**SE**	***t***	***P***
Intercept	15.34	0.17	92.34	<0.001	16.54	0.12	135.91	<0.001
Age	0.86	0.56	1.54	0.126	0.46	0.38	1.23	0.221
Age^2	-0.61	0.55	-1.12	0.266	-0.45	0.38	-1.18	0.239
Territory quality	0.09	0.18	0.48	0.629	0.02	0.12	0.13	0.896
Time of day (midday vs afternoon)	-0.28	0.20	-1.41	0.160	-0.12	0.13	-0.91	0.364
Tiime of day (morning vs afternoon)	-0.30	0.20	-1.52	0.132	-0.30	0.16	-1.91	0.058
Tarsus length	0.36	0.18	2.06	0.041	0.33	0.12	2.73	0.007
Helper (Y/N)	-0.16	0.23	-0.73	0.467	0.03	0.14	0.21	0.836
Random	**Variance**				**Variance**			
Individual ID	0.52		147 observations		0.40		213 observations	
Year	0.06		129 individuals		0.05		175 individuals	
Residual	0.48		14 years		0.28		14 years	

(a) Dominant females, (b) dominant males. Final models contained all main effects.

Supplementary Table 7. Changes in the likelihood that a subordinate helped in relation to the dominant's age and the subordinate's sex.

(a) Dominant female					(b) Dominant male			
**Variable**	**Estimate**	**SE**	***z***	***P***	**Estimate**	**SE**	***z***	***P***
Intercept	-0.97	0.44	-2.20	0.028	-1.14	0.37	-3.10	0.002
Dominant age	0.28	0.33	0.85	0.393	-0.03	0.31	-0.08	0.936
Territory quality	0.06	0.26	0.23	0.822	0.21	0.24	0.88	0.382
Subordinate sex (male)	-1.05	0.23	-4.61	<0.001	-0.94	0.21	-4.57	<0.001
Subordinate age (older)	1.66	0.22	7.45	<0.001	1.46	0.21	7.08	<0.001
Number of subordinates	0.09	0.21	0.45	0.652	-0.23	0.20	-1.18	0.240
Longevity	-0.09	0.31	-0.29	0.773	0.10	0.31	0.31	0.757
Dominant age * Subordinate sex	-1.29	0.48	-2.67	0.008	0.46	0.39	1.18	0.239
Random	**Variance**		694 observations		**Variance**		707 observations	
Group ID	<0.01		497 groups		<0.01		516 groups	
Individual ID	0.23		226 individuals		0.21		244 individuals	
Year	0.34		14 years		0.13		15 years	

(a) Dominant females, (b) dominant males. The analysis is similar to the analysis in Table 3, except that longevity of the dominant is included here to account for selective disappearance (i.e. individuals for which longevity was unknown were removed). Longevity was not significant and models that included longevity had a slightly higher AICc than the same models without this variable (ΔAICc dominant females = 1.98, ΔAICc dominant males = 1.92). Final models contained all main effects and significant interaction terms.
