# Supplementary material for: Breeders that receive help age more slowly in a cooperatively breeding bird
Source: Nat Commun. 2019 Mar 21;10:1301. doi: 10.1038/s41467-019-09229-3 (PMC6428877; doi:10.1038/s41467-019-09229-3)
Supplement: Supplementary file 3 — Reporting Summary [file 41467_2019_9229_MOESM3_ESM.pdf]

nature research

Corresponding author(s): Martijn Hammers

Last updated by author(s): Feb 28, 2019

Reporting Summary

Nature Research wishes to improve the reproducibility of the work that we publish. This form provides structure for consistency and transparency in reporting. For further information on Nature Research policies, see Authors & Referees and the Editorial Policy Checklist.

Statistics

For all statistical analyses, confirm that the following items are present in the figure legend, table legend, main text, or Methods section.

n/a	Confirmed	
☐	☒	The exact sample size (*n*) for each experimental group/condition, given as a discrete number and unit of measurement
☐	☒	A statement on whether measurements were taken from distinct samples or whether the same sample was measured repeatedly
☐	☒	The statistical test(s) used AND whether they are one- or two-sided<br>*Only common tests should be described solely by name; describe more complex techniques in the Methods section.*
☐	☒	A description of all covariates tested
☐	☒	A description of any assumptions or corrections, such as tests of normality and adjustment for multiple comparisons
☐	☒	A full description of the statistical parameters including central tendency (e.g. means) or other basic estimates (e.g. regression coefficient) AND variation (e.g. standard deviation) or associated estimates of uncertainty (e.g. confidence intervals)
☐	☒	For null hypothesis testing, the test statistic (e.g. *F*, *t*, *r*) with confidence intervals, effect sizes, degrees of freedom and *P* value noted<br>*Give P values as exact values whenever suitable.*
☒	☐	For Bayesian analysis, information on the choice of priors and Markov chain Monte Carlo settings
☐	☒	For hierarchical and complex designs, identification of the appropriate level for tests and full reporting of outcomes
☐	☒	Estimates of effect sizes (e.g. Cohen's *d*, Pearson's *r*), indicating how they were calculated

Our web collection on statistics for biologists contains articles on many of the points above.

Software and code

Policy information about availability of computer code

Data collection	Microsoft Excel 2010, Microsoft Access 2010
Data analysis	R version 3.2.5. R Package LME4 version version 1.1-12

For manuscripts utilizing custom algorithms or software that are central to the research but not yet described in published literature, software must be made available to editors/reviewers. We strongly encourage code deposition in a community repository (e.g. GitHub). See the Nature Research guidelines for submitting code & software for further information.

Data

Policy information about availability of data

All manuscripts must include a data availability statement. This statement should provide the following information, where applicable:

- Accession codes, unique identifiers, or web links for publicly available datasets
- A list of figures that have associated raw data
- A description of any restrictions on data availability

The data that support the findings of this study are available in figshare with the identifier doi:10.6084/m9.figshare.7751099

Field-specific reporting

Please select the one below that is the best fit for your research. If you are not sure, read the appropriate sections before making your selection.

☐ Life sciences ☐ Behavioural & social sciences ☒ Ecological, evolutionary & environmental sciences

For a reference copy of the document with all sections, see nature.com/documents/nr-reporting-summary-flat.pdf

Ecological, evolutionary & environmental sciences study design

All studies must disclose on these points even when the disclosure is negative.

Study description	Long-term longitudinal study of a wild bird population
Research sample	The indivdually-marked population (ca 320 individuals in 115 territories) of Seychelles warblers (Acrocephalus sechellensis) on Cousin Island was studied between 1995 and 2016.
Sampling strategy	All available data were used
Data collection	Each year during the main breeding season (June-September,) the population is intensively monitored by a team of fieldworkers and the long-term data collection is coordinated by David Richardson, Terry Burke, Hannah Dugdale and Jan Komdeur. Procedures include: catching and colour-banding unringed individuals, observations of colour-banded individuals, monitoring breeding success, taking blood samples, collecting data on helping behaviour, estimating insect availability.
Timing and spatial scale	The individually-marked population of Seychelles warblers (Acrocephalus sechellensis) on Cousin Island was studied between 1995 and 2016 during the main breeding season (June-September)
Data exclusions	We excluded the years 2000 – 2002 because fieldwork was limited in this period, with incomplete data on helping behaviour. In addition, we excluded 2004 because 58 individuals (both dominants and subordinates) were translocated to another island just before the main breeding season as part of a conservation programme, and 2005 because no territory quality data were collected in that year.
Reproducibility	We used all observational data the were available and did not perform experiments.
Randomization	All available data were used
Blinding	The data were collected as part of the long-term monitoring program of the Cousin Island Seychelles warbler population. Therefore, the people that collected the data were blind to the the specific research question that the data were to be used for. For the analyses, all available data were used.

Did the study involve field work? ☒ Yes ☐ No

Field work, collection and transport

Field conditions	The study was performed on a small tropical island with a stable high average annual temperature (27 degrees C), high humidity (80%) and high rainfall (ca 150mm per month)
Location	Cousin Island, Seychelles (29 ha); 4°20' S, 55°40' E
Access and import/export	The work was conducted with the permission of the Seychelles Bureau of Standards and the Seychelles Ministry of Environment, Energy and Climate Change and complied with all local ethical guidelines and regulations. Nature Seychelles provided permission to work on Cousin Island. The Seychelles Ministry of Environment, Energy and Climate Change provided export permits for samples.
Disturbance	Fieldworkers are clearly instructed on how to minimize their impact on wildlife and vegetation and training is provided to minimize disturbance during observations, catching and handling.

Reporting for specific materials, systems and methods

We require information from authors about some types of materials, experimental systems and methods used in many studies. Here, indicate whether each material, system or method listed is relevant to your study. If you are not sure if a list item applies to your research, read the appropriate section before selecting a response.

Materials & experimental systems

n/a	Involved in the study	
☒	☐	Antibodies
☒	☐	Eukaryotic cell lines
☒	☐	Palaeontology
☐	☒	Animals and other organisms
☒	☐	Human research participants
☒	☐	Clinical data

Methods

n/a	Involved in the study	
☒	☐	ChIP-seq
☒	☐	Flow cytometry
☒	☐	MRI-based neuroimaging

Animals and other organisms

Policy information about studies involving animals; ARRIVE guidelines recommended for reporting animal research

Laboratory animals	The study did not use laboratory animals
Wild animals	Wild Seychelles warblers (Acrocephalus sechellensis) of both sexes and of all ages in a natural population
Field-collected samples	Blood samples were stored in 100% ethanol and stored at room temperature and transported to and analysed in the lab at the University of Groningen (NL) and University of East Anglia (UK)
Ethics oversight	The work was conducted with the permission of the Seychelles Bureau of Standards and the Seychelles Ministry of Environment, Energy and Climate Change and complied with all local ethical guidelines and regulations. Nature Seychelles provided permission to work on Cousin Island.

Note that full information on the approval of the study protocol must also be provided in the manuscript.
